# Supplementary material for: FANCJ promotes PARP1 activity during DNA replication that is essential in BRCA1 deficient cells
Source: Nat Commun. 2024 Mar 23;15:2599. doi: 10.1038/s41467-024-46824-5 (PMC10960859; doi:10.1038/s41467-024-46824-5)
Supplement: Supplementary file 1 — Supplementary Information [file 41467_2024_46824_MOESM1_ESM.pdf]

Supplementary Figure 1

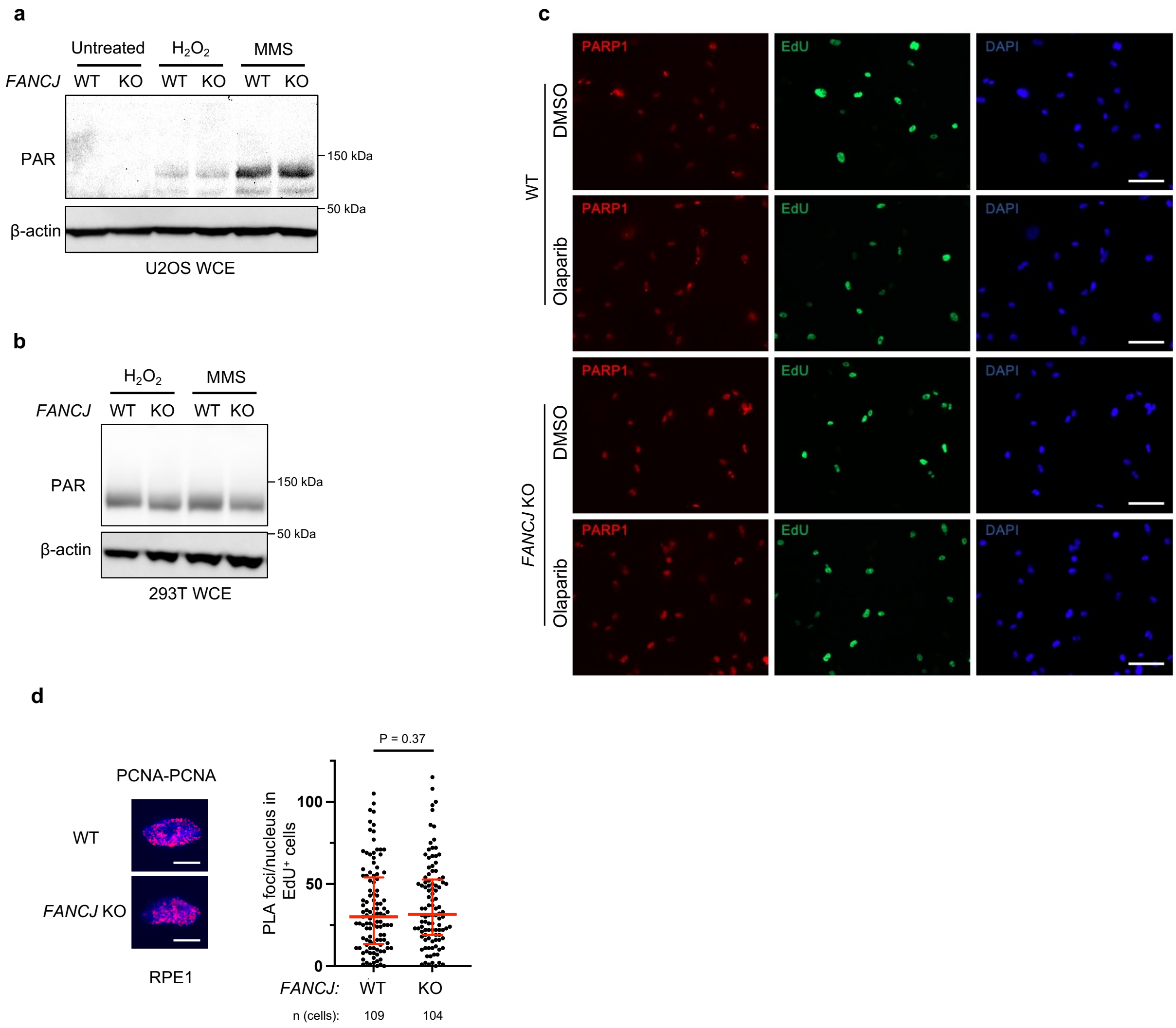

Supplementary Figure 1: FANCJ deficiency does not reduce PAR response to DNA damage.

a, b Representative WB from 2 independent experiments for PAR in the indicated cells under untreated growth conditions or following the treatment of DNA damage-inducing agents methyl methanesulfonate (MMS) and H_2O_2 for 40 min.

c Representative images related to Fig 1h. Scale bars, 50 µm

d PCNA-PCNA PLA assay in untreated RPE1 WT vs *FANCJ* KO cells with EdU incubated for 20 mins. Dot plot shows the number of foci per EdU^+ cell and the red bars represent median ± interquartile range from 3 independent experiments. Representative images shown with PLA foci in red, scale bars 10 µm. EdU-positive or EdU^+ cells were gated to identify positive EdU incorporation (S-phase). Statistical analysis according to two-tailed Mann-Whitney test.

All source data are provided as a Source Data file.

Supplementary Figure 2

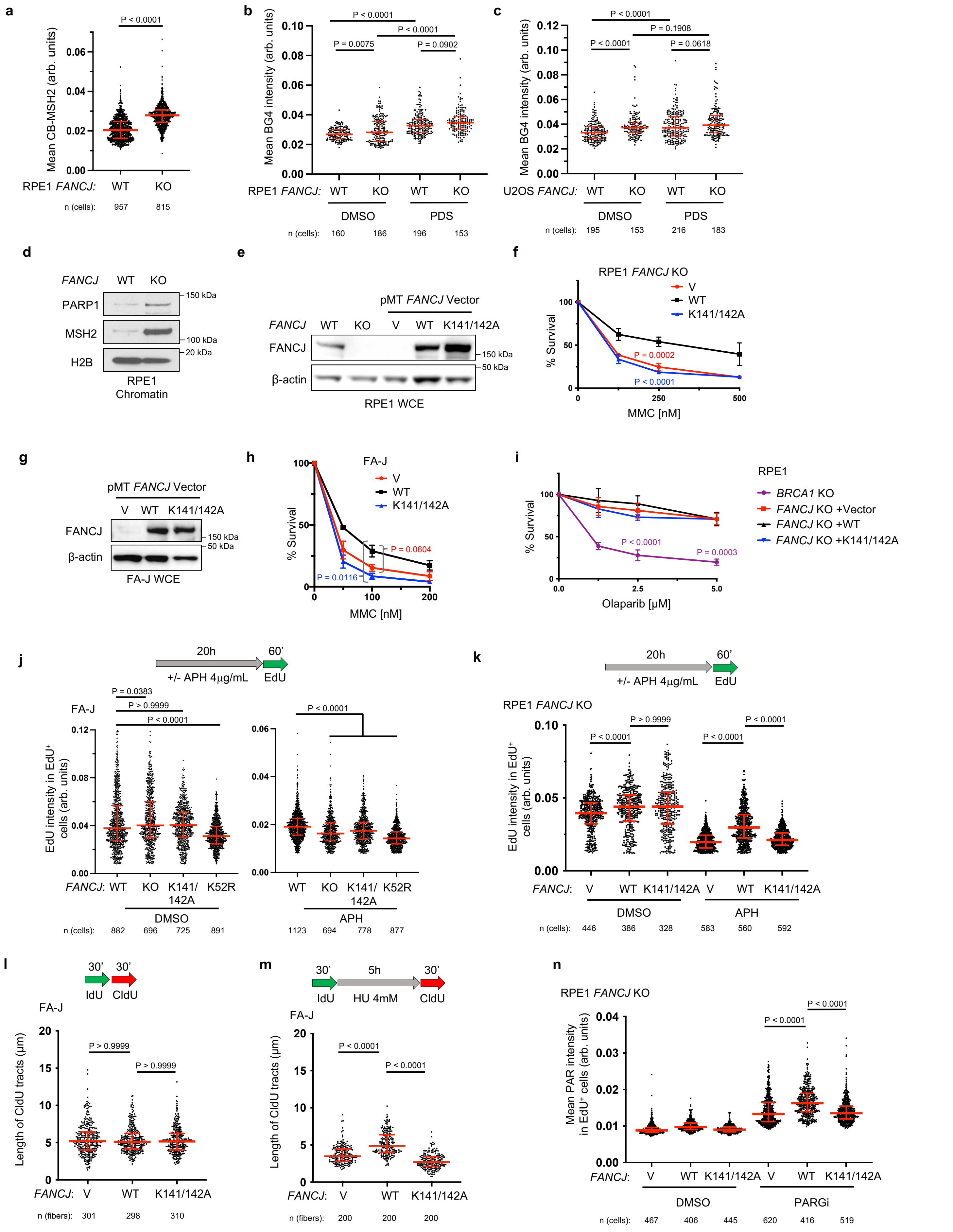

Supplementary Figure 2: Loss of *FANCJ* or FANCJ-MLH1 binding impairs RPA chromatin loading and replication restart.

a Quantification of chromatin-bound MSH2 (CB-MSH2) in RPE1 cells. Each dot represents one cell, red bars represent median ± interquartile range from 2 independent experiments. Statistical analysis according to two-tailed Mann-Whitney test.

b, c Mean BG4 fluorescence intensity in RPE1 and U2OS WT cells or *FANCJ* KO with or without 30 min 2 μM pyridostatin (PDS). Each dot represents one cell and red bars represent median ± interquartile range from 3 independent experiments. Statistical analysis according to Kruskal-Wallis test followed by Dunn's test.

d Representative WB from 2 independent experiments for chromatin bound PARP1 and MSH2 in RPE1 WT and *FANCJ* KO cells.

e, **f, g, h, i** Representative WB and cell survival assays validating the complement system in RPE1 FANCJ KO cells (**e**, **f**), FA-J cells (**g**, **h**), and with Olaparib (**i**) each from 3 independent experiments. Data represent the mean percentage ±SD of survival for each dot. Significance was determined by one-way ANOVA followed by Dunnett's test. P-value color matches sample in key, compares to WT.

j, k Quantification of mean EdU intensity from EdU^+ cells. EdU was incubated for 1hr after 20 hr DMSO or aphidicolin (APH, 4 μg/ml) treatment in the indicated cells: FANCJ mutant cells **j** and FANCJ K141/142A cells in complement systems **k**. Dots show mean EdU intensity with median ±interquartile range from 2 independent experiments. EdU^+ cells were gated to identify positive EdU incorporation. Statistical analysis according to Kruskal-Wallis test, followed by Dunn's test.

l, m Quantification of IdU-connected CldU tracts in indicated cells following (**l**) without or (**m**) with hydroxyurea (HU) treatment (4 mM, 5 h). Each dot represents 1 fiber; data are from 2 independent experiments. Red bars represent the median ±interquartile range. Statistical analysis according to Kruskal-Wallis test, followed by Dunn's test.

n Quantification of PAR intensity for indicated cells and complement systems. EdU^+ cells were gated according to positive EdU incorporation (S-phase). Each dot represents one cell from 2 independent experiments. Red bars represent the median ±interquartile range. Statistical analysis according to Kruskal-Wallis test, followed by Dunn's test.

All source data are provided as a Source Data file.

Supplementary Figure 3

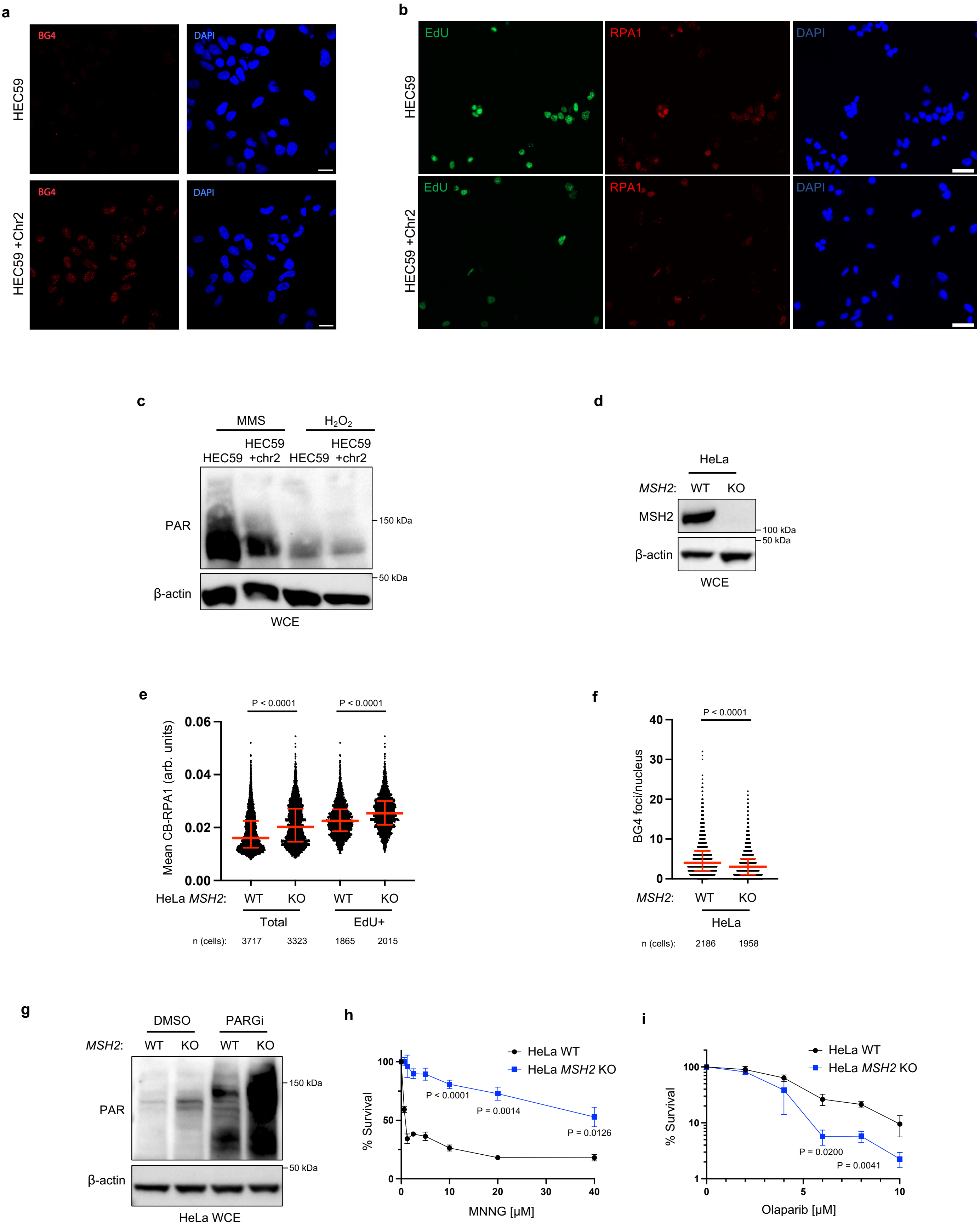

Supplementary Figure 3: MSH2 interferes with PARP1 activation.

a Representative images related to Fig 3b. Scale bars, 20 μm

b Representative images related to Fig 3c, d. Scale bars, 50 μm

c Representative WB from 3 independent experiments for the analysis of PAR formation in indicated cells treated with MMS or H_2O_2 for 40 min prior to harvesting.

d Representative WB from 2 independent experiments for analysis of the expression levels of the indicated proteins from whole cell lysates in untreated HeLa WT vs HeLa *MSH2* KO cells.

e Quantification of chromatin bound RPA1 for the indicated cells with EdU incubated for the 40 min. EdU-positive or EdU^+ cells were gated to identify positive EdU incorporation (S-phase). Each dot represents one cell from 3 independent experiments. Red bars represent the median ± interquartile range. Statistical analysis according to Kruskal-Wallis test, followed by Dunn's test.

f Quantification of G-quadruplexes (BG4 antibody) in untreated HeLa WT vs HeLa *MSH2* KO cells from 3 independent experiments. Red bars represent the median ± interquartile range. Statistical analysis according to two-tailed Mann-Whitney test.

g Representative WB from 1 independent experiment for PAR in the indicated cells treated with DMSO or PARGi for 40 min prior to harvesting.

h, i Cell survival assays under increasing concentrations of MNNG and Olaparib from 3 independent experiments. Data represent the mean percentage ± SD of survival for each dot. Significance was determined by unpaired t-test (two-tailed, unequal variance).

All source data are provided as a Source Data file.

Supplementary Figure 4

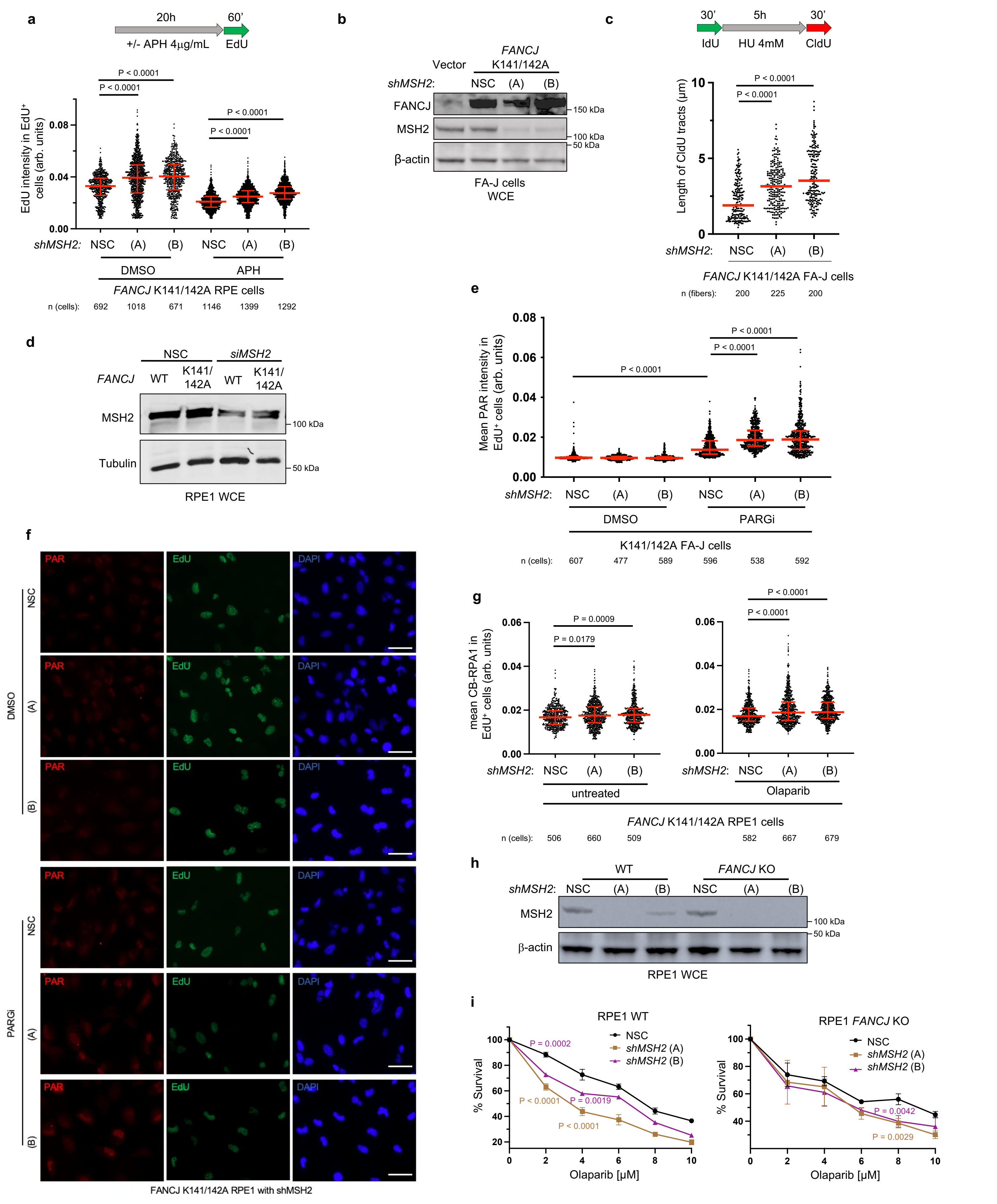

Supplementary Figure 4: The FANCJ-MLH1 interaction restricts MSH2.

a Quantification of mean EdU intensity from EdU$^+$ cells. EdU was incubated for 1hr after 20 hr DMSO or aphidicolin (APH, 4 μg/ml) treatment in the indicated RPE1 cells. EdU$^+$ cells were gated according to positive EdU incorporation. Each dot represents one cell from 2 independent experiments. Red bars represent the median ± interquartile range. Statistical analysis according to Kruskal-Wallis test, followed by Dunn's test.

b-c WB analysis and quantification of the DNA fiber assays for the length of IdU-connected CldU tracts interrupted by HU treatment (4 mM, 5 h) in FA-J complemented cells with *MSH2* depletion, Each dot represents 1 fiber; data are from 2 independent experiments. Red bars represent the median ± interquartile range. Statistical analysis according to Kruskal-Wallis test, followed by Dunn's test.

d Representative WB from 3 independent experiments for analysis of the expression levels of the indicated proteins from whole cell lysates in RPE1 WT and mutant cells.

e Quantification of PAR after 30min DMSO or PARGi (10 μM) treatment in the indicated EdU-positive FA-J cells. EdU$^+$ cells were gated according to positive EdU incorporation. Each dot represents one cell from 3 independent experiments. Red bars represent the median ± interquartile range. Statistical analysis according to Kruskal-Wallis test, followed by Dunn's test.

f Representative images related to Fig 4c. Scale bars, 50 μm

g Quantification of CB-RPA1 for the indicated cells for (left) untreated or (right) following the Olaparib (10μM, 6h), with EdU incubated for the final 40 min. EdU$^+$ cells were gated according to positive EdU incorporation. Each dot represents one cell from 3 independent experiments. Red bars represent the median ± interquartile range. Statistical analysis according to Kruskal-Wallis test, followed by Dunn's test.

h, i WB analysis and cell survival assays for the indicated RPE1 cells under increasing concentrations of olaparib. Data represent the mean percentage ± SD of survival for each dot from 3 representative experiments. Significance was determined by one-way ANOVA followed by Dunnett's test comparing NSC to *shMSH2* cells. P-value color matches sample in key, compares to NSC.

All source data are provided as a Source Data file.

Supplementary Figure 5

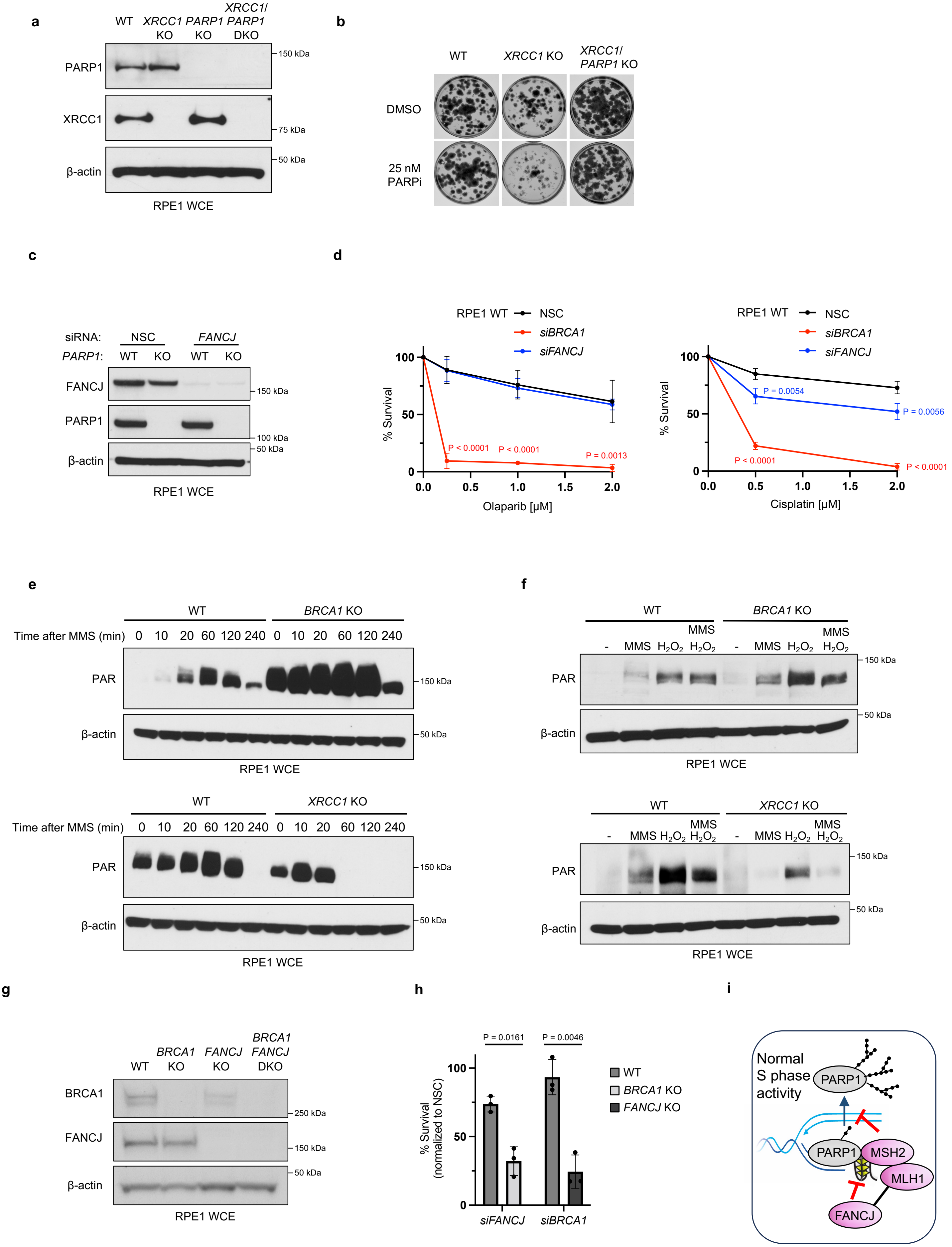

Supplementary Figure 5: PARP1 biology is distinct in BRCA1 and XRCC1 deficient cells.

a Representative WB from 3 independent experiments for analysis of the expression levels of the indicated proteins from whole cell lysates of untreated WT, *XRCC1* KO, *PARP1* KO and *XRCC1*/*PARP1* KO RPE1 cells.

b Representative clonogenic efficiency of the indicated stable RPE1 cell lines treated with PARPi from 3 independent experiments.

c Representative WB from 3 independent experiments for analysis of the expression levels of the indicated proteins from whole cell lysates from untreated WT vs *PARP1* KO RPE1 cells with or without *FANCJ* depletion.

d Cell survival assays for the indicated cells under increasing concentrations of Olaparib and Cisplatin treatment. Data represent the mean percentage ± SD of survival for each dot from three independent experiments. Significance was determined by one-way ANOVA followed by Dunnett's test, P-value color matches sample in key, compares to NSC.

e Representative WB from 3 independent experiments for PAR from whole cell lysates in the indicated isogenic cells following MMS (0.1mg/ml) treatment at different time points for 0-240min.

f Representative WB from 2 independent experiments for PAR from whole cell lysates in the indicated isogenic cells following MMS (0.1mg/ml, 60 min), H_2O_2 (2mM, 10 min), or combined treatment (MMS 0.1mg/ml 60 min followed by H_2O_2 2mM, 10min).

g Representative WB from 2 independent experiments for analysis of the expression levels of the indicated proteins from whole cell lysates of RPE1 WT, *BRCA1* KO, *FANCJ* KO and *BRCA1*/*FANCJ* double KO (DKO) cells.

h Quantification of clonogenic survival assays of the indicated RPE1 cell lines and knockdown reagents. Dots represent each individual experiment while the top of the bar indicates the mean percentage ± SD from 3 independent experiments. Significance determined by one-way ANOVA (unequal variance) followed by Dunnett's test.

i Model that FANCJ dismantles replisome-associated MSH2-bound G4s through its MLH1 binding and resolves G4 structures to activate and release PARP1 from chromatin.

All source data are provided as a Source Data file.

Oligos used to generate FANCJ mutants:

Gene variant	PCR primers	gRNA	ssODN
K52R	5'ATTCTGGCACA GATTCCTCTT 5'CCATGCCTGGC TAATTGATTG	5'AAGCTTTTTC CACTTCCTGT	ACTACTTACCACTAAGAGATT GTTGCCATGCTAAAGCAGAA CAAAGTAAGGCTAAGCTCCG TCCACTTCCTGTTGGACTCTC CAACAAACAATGTTGCTTGCT GTTTAATCCTCTGAGAA
K141/ 142A	5'AGAAGGAGCTT TCAGGATTATGG 5'CCATGCAGTTT CACTTGAACG	5'AAAGTTATCT GCTAAGAAAC	TAAATTACTTATATAAGACTC CCCTGAAAAAACCACTCTGG CTGCAAAGTTATCTGCTGCG GCACAGGCATCCATATACAG AGATGAAAATGATGATTTTCA AGTAGAGAAGAAAAGAAT

Supplementary table 1: PCR primers used to validate *FANCJ* knock-in clones and oligos used to edit *FANCJ* alleles. Guide RNAs (gRNA) used for targeting and the associated repair template single-stranded oligodeoxynucleotides (ssODN).
