# Supplementary material for: FANCJ promotes PARP1 activity during DNA replication that is essential in BRCA1 deficient cells
Source: Nat Commun. 2024 Mar 23;15:2599. doi: 10.1038/s41467-024-46824-5 (PMC10960859; doi:10.1038/s41467-024-46824-5)
Supplement: Supplementary file 3 — Reporting Summary [file 41467_2024_46824_MOESM3_ESM.pdf]

Corresponding author(s): Sharon Cantor
Last updated by author(s): 2/13/2024

Reporting Summary

Nature Portfolio wishes to improve the reproducibility of the work that we publish. This form provides structure for consistency and transparency in reporting. For further information on Nature Portfolio policies, see our Editorial Policies and the Editorial Policy Checklist.

Statistics

For all statistical analyses, confirm that the following items are present in the figure legend, table legend, main text, or Methods section.

n/a	Confirmed	
☐	☒	The exact sample size (n) for each experimental group/condition, given as a discrete number and unit of measurement
☐	☒	A statement on whether measurements were taken from distinct samples or whether the same sample was measured repeatedly
☐	☒	The statistical test(s) used AND whether they are one- or two-sided<br>*Only common tests should be described solely by name; describe more complex techniques in the Methods section.*
☐	☒	A description of all covariates tested
☐	☒	A description of any assumptions or corrections, such as tests of normality and adjustment for multiple comparisons
☐	☒	A full description of the statistical parameters including central tendency (e.g. means) or other basic estimates (e.g. regression coefficient) AND variation (e.g. standard deviation) or associated estimates of uncertainty (e.g. confidence intervals)
☐	☒	For null hypothesis testing, the test statistic (e.g. F, t, r) with confidence intervals, effect sizes, degrees of freedom and P value noted<br>*Give P values as exact values whenever suitable.*
☒	☐	For Bayesian analysis, information on the choice of priors and Markov chain Monte Carlo settings
☒	☐	For hierarchical and complex designs, identification of the appropriate level for tests and full reporting of outcomes
☒	☐	Estimates of effect sizes (e.g. Cohen's d, Pearson's r), indicating how they were calculated

Our web collection on statistics for biologists contains articles on many of the points above.

Software and code

Policy information about availability of computer code

Data collection	Microscopy data were collected by a Ziess Axio-Scope fluorescent microscope with ZEN blue 2.5 pro software with a monochrome camera (channels were subsequently pseudo-colored for data representation) or a Zeiss LSM 710 confocal microscope . Western blot data were imaged by Bio-Rad chemiluminescence imaging system or a Kodak X-OMAT 2000A Film Processor. Luminescence was read by Beckman Coulter DTX 880 Multimode Detector.
Data analysis	Representative immunofluorescence images were adjusted by Fiji (ImageJ, 2.9.0). The results (e.g. intensity, foci) of the immunofluorescence assays were analyzed by CellProfiler v3.1.5 and v4.2.1 as noted in the methods section. Western blot analysis was performed in Fiji v2.1.0. Data were plotted and analyzed by GraphPad Prism v9.4.1 and v9.2.0 as noted in the methods section.

For manuscripts utilizing custom algorithms or software that are central to the research but not yet described in published literature, software must be made available to editors and reviewers. We strongly encourage code deposition in a community repository (e.g. GitHub). See the Nature Portfolio guidelines for submitting code & software for further information.

Data

Policy information about availability of data

All manuscripts must include a data availability statement. This statement should provide the following information, where applicable:

- Accession codes, unique identifiers, or web links for publicly available datasets
- A description of any restrictions on data availability
- For clinical datasets or third party data, please ensure that the statement adheres to our policy

Source data are provided as a Source Data file. Unprocessed blots and microscopy images are available online or through lead contact upon request (https://figshare.com/s/5052bf424786dba81c33 or DOI: 10.6084/m9.figshare.24280162). All materials associated with this study are available upon request from the lead contact. Further information and requests for resources and reagents should be directed to and will be fulfilled by the Lead Contact, Sharon Cantor (sharon.cantor@umassmed.edu).

Research involving human participants, their data, or biological material

Policy information about studies with human participants or human data. See also policy information about sex, gender (identity/presentation), and sexual orientation and race, ethnicity and racism.

Reporting on sex and gender	No human research.
Reporting on race, ethnicity, or other socially relevant groupings	No human research.
Population characteristics	No human research.
Recruitment	No human research.
Ethics oversight	No human research.

Note that full information on the approval of the study protocol must also be provided in the manuscript.

Field-specific reporting

Please select the one below that is the best fit for your research. If you are not sure, read the appropriate sections before making your selection.

☒ Life sciences ☐ Behavioural & social sciences ☐ Ecological, evolutionary & environmental sciences

For a reference copy of the document with all sections, see nature.com/documents/nr-reporting-summary-flat.pdf

Life sciences study design

All studies must disclose on these points even when the disclosure is negative.

Sample size	No statistical methods were used to pre-determine sample size. Sample sizes (at least 3 independent experimental replicates in most experiments, unless indicated otherwise) were chosen based on the standard practices of the field and by recently published articles with similar methods. Sample sizes were kept similar within experiments and the number of individual data points noted in the legends or figures. Data were collected in a manner to allow for robust statisical analysis and power.
Data exclusions	No data were excluded.
Replication	All the results were successfully replicated in at least 3 independent experiments unless otherwise noted. Details of Individual experiment are included in figure legends and methods respectively.
Randomization	Randomization is not typically applicable when studying specific biological events from known genetic cell line models in order to assess the biological consequences of gene loss in a specific genetic background. However, for the immunofluorescence, proximity ligation assay and the DNA fiber analysis, all the samples, cells and observation windows were randomly selected. For other experiments, data are collected from the response of the whole assay culture (survival, western blotting), therefore randomization between cells within a population are representative of the average of population. To control for this, multiple independent experiments were performed.
Blinding	Blind scoring of data was not performed. However, different co-authors examined the data and repeated most of the assays over a period of time (months/years) to ensure robust reproducibility of the data.

Reporting for specific materials, systems and methods

We require information from authors about some types of materials, experimental systems and methods used in many studies. Here, indicate whether each material, system or method listed is relevant to your study. If you are not sure if a list item applies to your research, read the appropriate section before selecting a response.

Materials & experimental systems

n/a	Involved in the study
☐	☒ Antibodies
☐	☒ Eukaryotic cell lines
☒	☐ Palaeontology and archaeology
☒	☐ Animals and other organisms
☒	☐ Clinical data
☒	☐ Dual use research of concern
☒	☐ Plants

Methods

n/a	Involved in the study
☒	☐ ChIP-seq
☒	☐ Flow cytometry
☒	☐ MRI-based neuroimaging

Antibodies

Antibodies used

Primary antibodies for western blot analysis included anti-FANCJ (E67 from Cantor lab), anti-beta-Actin (Sigma-Aldrich A1978), anti-PAR (R&D Systems 4335-MC-100), anti-PAR (poly-ADP-ribose binding reagent, Millipore Sigma MABE1031), anti-PARP1 (Abcam ab227244), anti-H2B (Cell Signaling Technology 8135), anti-MSH2 (Abcam ab52266), anti-XRCC1 (Abcam ab134056), anti-BRCA1 (Santa Cruz, D-9, sc6954), anti-Tubulin (Abcam, ab6160). Secondary antibodies for western blot analysis included ECL anti-rabbit IgG, HRP-linked whole antibody (from donkey, NA934) and ECL anti-mouse IgG, HRP-linked F(ab')$_2$ fragment (from sheep, NA9310). Primary antibodies for immunofluorescence based analysis included anti-PAR (Trevigen 4336-BPC-100), anti-PARP1 (Abcam ab227244), anti-MSH2 (Abcam ab52266), anti-RPA1 (Cell Signaling Technology 2267), anti-PCNA (Abcam ab18197), anti-PCNA (Abcam ab29), BG4 antibody (in-house from Day lab), anti-FLAG (Cell Signaling Technology, 14793S), anti-FLAG (Sigma, F1804) anti-BrdU (Becton Dickinson 347580), anti-BrdU (Abcam 6326). Secondary antibodies for immunofluorescence based analysis included Alexa Fluor 488 goat anti-Mouse (Thermo Fisher Scientific A-11001), Alexa Fluor 568 goat anti-Rabbit (Thermo Fisher Scientific A-11011), Alexa Fluor 594 goat anti-Rabbit (Thermo Fisher Scientific A-11012).

Validation

Antibodies were validated as stated on the suppliers' datasheets or cited studies below:

anti-FANCJ (E67):
Originated and validated for WB in Cantor et al., PNAS 2004, PMID: 14983014;

anti-β-Actin (Sigma-Aldrich, AC-15, A1978):
https://www.sigmaaldrich.com/US/en/product/sigma/a1978;

anti-PAR (R&D Systems, 10HA, 4335-MC-100):
https://www.rndsystems.com/products/par-padpr-antibody-10ha_4335-mc-100

anti-PAR (poly-ADP-ribose binding reagent, Millipore Sigma MABE1031): https://www.emdmillipore.com/US/en/product/Anti-poly-ADP-ribose-binding-reagent,MM_NF-MABE1031

anti-PARP1 (Abcam, ab227244):
https://www.abcam.com/products/primary-antibodies/parp1-antibody-ab227244.html;

anti-H2B (Cell Signaling Technology, 8135): https://www.cellsignal.com/product/productDetail.jsp?productId=8135;

anti-MSH2 (Abcam, 3A2B8C, ab52266): https://www.abcam.com/products/primary-antibodies/msh2-antibody-3a2b8c-ab52266.html;

anti-XRCC1 (Abcam, EPR4389(2)), ab134056): https://www.abcam.com/products/primary-antibodies/xrcc1-antibody-epr43892-ab134056.html;

anti-BRCA1 (Santa Cruz, D-9, sc6954): https://www.scbt.com/p/brca1-antibody-d-9;

anti-Tubulin (Abcam, YL1/2, ab6160): https://www.abcam.com/products/primary-antibodies/tubulin-antibody-yl12-loading-control-ab6160.html;

anti-PAR (Trevigen, 4336-BPC-100): https://www.bio-techne.com/p/antibodies/anti-par-polyclonal-antibody-rabbit_4336-bpc-100;

anti-RPA1 (Cell Signaling Technology, 2267): https://www.cellsignal.com/products/primary-antibodies/rpa70-rpa1-antibody/2267;

anti-PCNA (Abcam, ab18197): https://www.abcam.com/products/primary-antibodies/pcna-antibody-ab18197.html;

anti-PCNA (Abcam, PC10, ab29): https://www.abcam.com/products/primary-antibodies/pcna-antibody-pc10-ab29.html;

anti-BG4: The scFV BG4 was produced and validated in-house with similar methods described by the inventing Balasubramanian lab (Biffi et al. Nature Chemistry 2013), PMID: 23422559.
Stock concentration: 0.164 µg/µL

anti-FLAG (Cell Signaling Technology, D6W5B, 14793S): https://www.cellsignal.com/product/productDetail.jsp?productId=14793;

anti-FLAG (Sigma, M2, F1804): https://www.sigmaaldrich.com/US/en/product/sigma/f1804;
anti-BrdU (Becton Dickinson, B44, 347580): https://www.bdbiosciences.com/en-us/products/reagents/flow-cytometry-reagents/clinical-discovery-research/single-color-antibodies-ruo-gmp/purified-mouse-anti-brdu.347580;

anti-BrdU (Abcam, BU1/75 (ICR1), ab6326): https://www.abcam.com/products/primary-antibodies/brdu-antibody-bu175-icr1-proliferation-marker-ab6326.html;

ECL anti-mouse IgG, HRP-linked F(ab')$_2$ fragment (from sheep, NA9310): https://www.cytivalifesciences.com/en/us/shop/protein-analysis/blotting-and-detection/blotting-standards-and-reagents/amersham-ecl-hrp-conjugated-antibodies-p-06260;

ECL anti-rabbit IgG, HRP-linked whole antibody (from donkey, NA934) and ECL anti-mouse IgG, HRP-linked F(ab')$_2$ fragment (from sheep, NA9310): https://www.cytivalifesciences.com/en/us/shop/protein-analysis/blotting-and-detection/blotting-standards-and-reagents/amersham-ecl-hrp-conjugated-antibodies-p-06260;

Alexa Fluor 488 goat anti-Mouse (Thermo Fisher Scientific A-11001): https://www.thermofisher.com/antibody/product/Goat-anti-Mouse-IgG-H-L-Cross-Adsorbed-Secondary-Antibody-Polyclonal/A-11001;

Alexa Fluor 568 goat anti-Rabbit (Thermo Fisher Scientific A-11011): https://www.thermofisher.com/antibody/product/Goat-anti-Rabbit-IgG-H-L-Cross-Adsorbed-Secondary-Antibody-Polyclonal/A-11011;

Alexa Fluor 594 goat anti-Rabbit (Thermo Fisher Scientific A-11012): https://www.thermofisher.com/antibody/product/Goat-anti-Rabbit-IgG-H-L-Cross-Adsorbed-Secondary-Antibody-Polyclonal/A-11012;

Eukaryotic cell lines

Policy information about cell lines and Sex and Gender in Research

Cell line source(s)

Human RPE1-hTERT TP53-/- (WT, parental) and BRCA1 KO cells are from Dr. Daniel Durocher. The 293T and U2OS derived cell lines have been used before and their generation was described previously in Cong et al, Mol Cell, 2021;81(15):3128-3144.e7. The XRCC1/PARP1 single KOs, DKO and matched isogenic control RPE1 cells were obtained from the Dr. Keith Caldecott lab and used in Demin et al, Mol Cell, 2021;81(14):3018-3030.e5. The HeLa MSH2 KO and matched isogenic HeLa WT cells, and the human endometrial HEC59 and HEC59+chr2 cell lines, were obtained from Dr. Christopher Heinen lab. The FA-J (EUFA30-F) cells were from Dr. Hans Joenje.

Authentication

Cell lines were validated by immunoblot and/or genomic sequencing for the desired protein/gene. Expected or previously reported phenotypes could also be reproduced.

Mycoplasma contamination

All cell lines tested negative for mycoplasma and were routinely tested for mycoplasma contamination before use with the Lonza MycoAlert Mycoplasma Detection Kit.

Commonly misidentified lines
(See ICLAC register)

No commonly misidentified cell lines were used in this study.

Plants

Seed stocks

Report on the source of all seed stocks or other plant material used. If applicable, state the seed stock centre and catalogue number. If plant specimens were collected from the field, describe the collection location, date and sampling procedures.

Novel plant genotypes

Describe the methods by which all novel plant genotypes were produced. This includes those generated by transgenic approaches, gene editing, chemical/radiation-based mutagenesis and hybridization. For transgenic lines, describe the transformation method, the number of independent lines analyzed and the generation upon which experiments were performed. For gene-edited lines, describe the editor used, the endogenous sequence targeted for editing, the targeting guide RNA sequence (if applicable) and how the editor was applied.

Authentication

Describe any authentication procedures for each seed stock used or novel genotype generated. Describe any experiments used to assess the effect of a mutation and, where applicable, how potential secondary effects (e.g. second site T-DNA insertions, mosiacism, off-target gene editing) were examined.
